# SyntenyViewer: a comparative genomics-driven translational research tool

**DOI:** 10.1093/database/baad027

**Published:** 2023-05-09

**Authors:** Raphael Flores, Cécile Huneau, Laura Burlot, Mathilde Lainé, Erik Kimmel, Cyril Pommier, Michael Alaux, Anne-Françoise Adam-Blondon, Caroline Pont, Hadi Quesneville, Jerome Salse

**Affiliations:** Université Paris-Saclay, INRAE, URGI, Versailles 78026, France; Plant Bioinformatics Facility, Université Paris-Saclay, INRAE, BioinfOmics, Versailles 78026, France; UCA, INRAE, GDEC 1095, Clermont-Ferrand 63000, France; Université Paris-Saclay, INRAE, URGI, Versailles 78026, France; Université Paris-Saclay, INRAE, URGI, Versailles 78026, France; Université Paris-Saclay, INRAE, URGI, Versailles 78026, France; Plant Bioinformatics Facility, Université Paris-Saclay, INRAE, BioinfOmics, Versailles 78026, France; Université Paris-Saclay, INRAE, URGI, Versailles 78026, France; Plant Bioinformatics Facility, Université Paris-Saclay, INRAE, BioinfOmics, Versailles 78026, France; Université Paris-Saclay, INRAE, URGI, Versailles 78026, France; Plant Bioinformatics Facility, Université Paris-Saclay, INRAE, BioinfOmics, Versailles 78026, France; Université Paris-Saclay, INRAE, URGI, Versailles 78026, France; Plant Bioinformatics Facility, Université Paris-Saclay, INRAE, BioinfOmics, Versailles 78026, France; UCA, INRAE, GDEC 1095, Clermont-Ferrand 63000, France; Université Paris-Saclay, INRAE, URGI, Versailles 78026, France; Plant Bioinformatics Facility, Université Paris-Saclay, INRAE, BioinfOmics, Versailles 78026, France; UCA, INRAE, GDEC 1095, Clermont-Ferrand 63000, France; Université Paris-Saclay, INRAE, URGI, Versailles 78026, France; Plant Bioinformatics Facility, Université Paris-Saclay, INRAE, BioinfOmics, Versailles 78026, France

## Abstract

SyntenyViewer is a public web-based tool relying on a relational database available at https://urgi.versailles.inrae.fr/synteny delivering comparative genomics data and associated reservoir of conserved genes between angiosperm species for both fundamental (evolutionary studies) and applied (translational research) applications. SyntenyViewer is made available for (i) providing comparative genomics data for seven major botanical families of flowering plants, (ii) delivering a robust catalog of 103 465 conserved genes between 44 species and inferred ancestral genomes, (iii) allowing us to investigate the evolutionary fate of ancestral genes and genomic regions in modern species through duplications, inversions, deletions, fusions, fissions and translocations, (iv) use as a tool to conduct translational research of key trait-related genes from model species to crops and (v) offering to host any comparative genomics data following simplified procedures and formats

**Database URL** https://urgi.versailles.inrae.fr/synteny

Key points

- SyntenyViewer is a web resource to perform comparative genomics in plants;
- SyntenyViewer allows access to expertised data and to download novel analyses;
- SyntenyViewer provides methods, scripts, documents and procedures to generate comparative genomics data.

## Introduction

Flowering plants, or angiosperms, emerged some 120–250 million years ago, depending on the dating approach (1–3), to rapidly diversify into 350 000 species alive today (4–7). These species are divided into two main groups, the monocots and eudicots, which, respectively, account for 20% and 75% of the plant diversity characterized to date (6). Cost reduction and technical improvements in sequencing technology make increasingly available public high-quality plant genome sequences offering the opportunity to conduct in-depth comparative genomics (8). Knowledge on gene functions in relation to traits and processes as well as genome evolutionary dynamics is gained from accurate comparative genomics investigation. In that regard, several public tools are available to query comparative genomics data between plant genomes such as PLAZA (9), Gramene (10), Ensembl (11), CoGe (12) and Genomicus (13). However, methodologies can differ in defining conserved genes between species, making it particularly difficult to take into account recurrent whole-genome duplication (WGD) events in plant paleohistory, leading to artefactual identification of conserved genes (14). All extant species are either ancient (paleo-) or modern (neo-) polyploids derived from either the doubling of a single parental genome (autopolyploidy, AA deriving AAAA) or the hybridization of two parental genomes (allopolyploidy, AA × BB deriving AABB) (15). Consequently, all extant genomes may contain more than one copy of each ancestral gene. However, the accepted subgenome fractionation mechanism following polyploidization and consisting in the bias erosion of the ancestral gene content between the two parental genomes in the newly formed polyploid species, and then leading to least fractionated (LF) and most fractionated (MF) genomic fractions, leads to the progressive deletion of duplicated genes over time (16). Then, recurrent polyploidization–fractionation cycles in the course of plant evolution make the precise identification of conserved (orthologs) and duplicated (paralogs) genes in plant comparative genomics studies difficult. This article presents SyntenyViewer, a web-based tool hosting expertised synteny relationships between angiosperm genomes through the reconstruction of ancestral genomes (17), and discusses potential uses of the delivered catalog of conserved genes for evolutionary studies as well translational research investigation.

## Materials and methods

### Synteny inference through ancestral genome reconstruction

**Figure 1.**
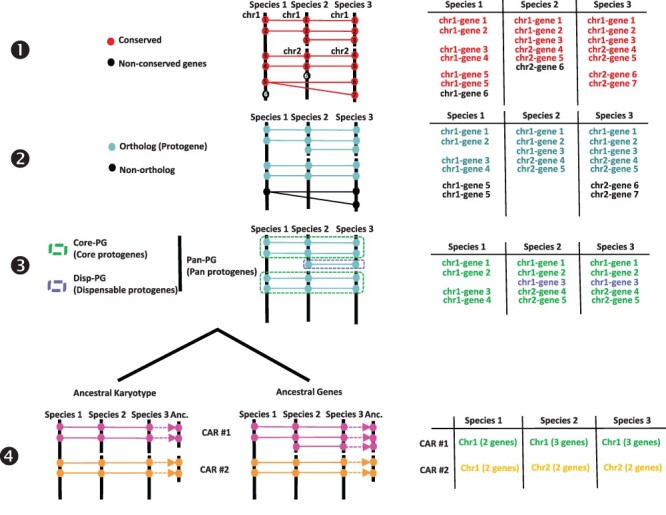
Procedure for reconstructing ancestral karyotypes. Ancestral genomes are inferred from (see the Materials and methods section) conserved genes (Step 1), orthologous relationships (Step 2), SBs (Step 3) and CARs (Step 4), to provide the best scenario explaining the transition between ancestral and modern genomes. Types of tabular files derived from each step are illustrated at the right to help readers to properly follow the procedure (described in and adapted from (20)).

From an ancestral (possibly extinct) genome that evolved into different extant species through speciation and distinct chromosome shuffling events (fusions, fissions, inversions and translations), each of the ancestral chromosomes will derive a subset of extant chromosomal regions sharing synteny. Following this evolutionary evidence when reconstructing ancestral karyotypes *in silico*, comparative genomics of modern genomes should produce genomic fragments showing independent (non-shared) syntenic blocks, referred to as conserved ancestral regions (CARs), which are considered as ancestral chromosomes in the inferred ancestral karyotype. We proposed (14, 17–20) a four-step method to infer ancestral genomes from BLAST-based comparison of modern genomes (Figure 1). The genes (protein sequences) from the investigated precise are compared using BLASTP with thresholds for cumulative identity percentage (CIP) ≥ 50% and cumulative alignment length percentage blast parameter (CALP) ≥ 50%, which deliver conserved genes between the investigated species using the following formulas:$CIP\; = \;\sum \;nb\;ID\;by\;\left( {\frac{{HSP}}{{AL}}} \right)\; \times 100$, where CIP corresponds to the cumulative percent of sequence identity observed for all the high-scoring pairs (HSPs) divided by the cumulative aligned length (AL), which corresponds to the sum of all HSP lengths; and $CALP\; = \;\frac{{AL}}{{Query\;length}}$, where CALP is the sum of the HSP lengths (AL) for all HSPs divided by the length of the query sequence. With these parameters, BLAST produces the highest cumulative percentage identity over the longest cumulative length, thereby increasing stringency in defining conserved genes between two genome sequences. From the previous BLAST comparison, the first step consists in retaining conserved genes. The second step consists in retaining single-copy orthologs and removing species-specific and tandem duplicates. This step consists in extracting one-to-one gene relationships (or 1–*n* relationships for *n* WGD events) between species from the Step 1 output file. The third step consists in clustering or chaining groups of conserved genes into synteny blocks (SBs), which reveal core protogenes (Core-PGs) conserved in all the investigated species or dispensable PGs (Disp-PGs) between conserved genes in a subset (at least two) of the investigated species. This step consists of extracting all combinations of chromosome-to-chromosome relationships (for SBs sharing more than five orthologous genes) from the Step 2 output file. In the fourth step, SBs from the previous output file are then merged into ancestral protochromosomes (also referred to as CARs). This step consists of defining independent groups of SBs sharing synteny between the modern species investigated. When the ancestral karyotype has been defined in its chromosome structure, conserved genes beyond one-to-one gene relationships between species (from Step 1) can be included in each protochromosome.

### SyntenyViewer database interface

**Figure 2.**
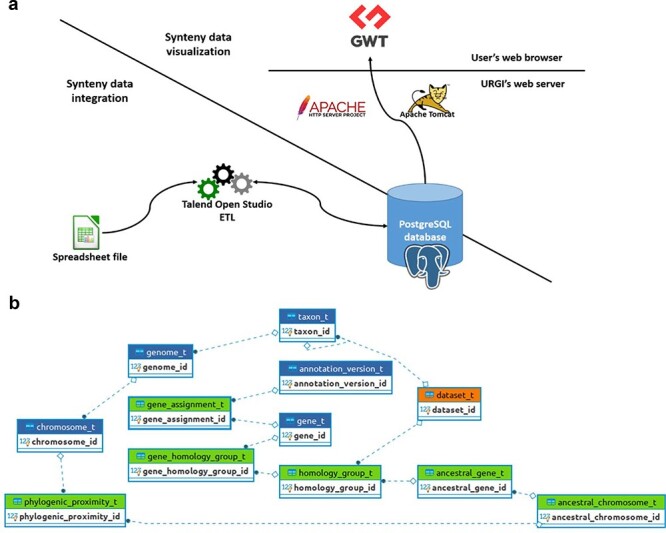
SyntenyViewer data processing and database description. (a) Illustration of theSyntenyViewer architecture including the data integration step into a PostgreSQL instance, and the data visualization based on GWT powered by Apache Tomcat and Apache HTTP server. (b) Illustration of the SyntenyViewer database model with for each box a table named with its primary key term (referenced below). Tables colored in green store data in an append only fashion when a new synteny dataset is submitted, blue tables contain new data as well as data shared between different datasets, orange table stores new data as well as updated data from a previously inserted dataset (i.e. ‘dataset_t’ that handles several versions of a dataset: a new tuple is inserted for Version 2 of Dataset A, while the tuple with Version 1 is marked obsolete). Some technical relationships between tables have been hidden for clarity. The database is structured below.

SyntenyViewer is a tool relying on a relational database (DB), aiming at displaying and making publicly available the previously described comparative genomics data at https://urgi.versailles.inrae.fr/synteny. The Java web application uses the Google Web Toolkit (GWT) framework for graphical dynamic web content processing. On the back end, the web server uses Apache HTTP and Apache Tomcat, while the DB management system relies on a PostgreSQL 9.6 instance to store the data ensuring referential integrity (Figure 2).

***‘*** Table dataset_t’: this contains information about each dataset (name, version, DOI). ‘Table gene_assignment_t’: this contains genomic information of a gene (position, strand, phase…). ‘Table homology_group_t’: this represents a group of conserved genes. ‘Table gene_homology_group_t’: this makes the link between a gene and its orthologous groups.‘Table ancestral_gene_t’: this contains the information of PGs.‘Table ancestral_chromosome_t’: this stores the reconstructed protochromosomes‘Table phylogenic_proximity_t’: this allows us to store the evolutionary distance between a reconstructed protochromosome and its descendant (modern) chromosomes

## Results

### Genome synteny between angiosperm species and within major botanical families

Genome synteny has been obtained following a four-step method consisting in the identification of conserved genes (Step 1), orthologous relationships (Step 2), SBs (Step 3) and CARs (Step 4), Figure 1. Following this methodology, SyntenyViewer delivers published comparative genomics data (listed in Table 1) obtained for the two major angiosperm families with the grasses within the monocots [ancestral grass karyotype (AGK) with 12 protochromosomes and 16 560 PGs (21, 22)] and the eudicots [ancestral eudicot karyotype (AEK) with 21 chromosomes and 10 286 PGs (23)]. SyntenyViewer also provides published comparative genomics data for angiosperm lineages of agronomical interest such as *Rosaceae* [ancestral *Rosaceae* karyotype (ARK) with nine protochromosomes and 8861 PGs (24, 25)], *Brassicaceae* [ancestral *Brassicaceae* karyotype (ABK) with eight protochromosomes and 20 037 PGs (26, 27)], *Cucurbitaceae* [ancestral *Cucurbitaceae* karyotype (ACuK) with 22 protochromosomes and 17 969 PGs (28, 29)], legumes [ancestral legume karyotype (ALK) with 16 protochromosomes and 13 181 PGs (30–32)] and *Solanaceae* [ancestral *Solanaceae* karyotype (ASK) with 17 protochromosomes and 17 879 PGs (33)]. All datasets are licensed under the Open Licence Version 2.0 (CC-BY-compatible) as described in the ‘Terms’ tab of each dataset from the French national scientific data repository Recherche Data Gouv (RDG) https://recherche.data.gouv.fr/en. For example, see the Terms tab of ‘PlantSyntenyViewer Solanaceae submission file’ where the license is also prompted when a user attempts to download the associated file (Table 1). Genome version and information are available at the RDG portal (see references).

**Table 1.** Ancestral plant genomes

Family	Dating	Ancestor	Chromosome number	Gene number	Species	Data accessed
Eudicots	87–109	AEK (post-γ)	21	10286	Papaya, Arabidopsis thaliana, cacao, soybean, lotus, apple, strawberry, poplar, grape	Murat *et al.* 2015 (23)
Grasses	65–81	AGK (post-ρ)	12	16560	Rice, *Brachypodium*, barley, wheat, setaria, sorghum, maize	Murat *et al.* 2010 (21), 2014 (22)
*Brassicaceae*	27–40	ABK (post-α/β)	8	20037	*Arabidopsis thaliana, Arabidopsis lyrata, Capsella rubella, Brassica rapa, Thellungiella parvula*	https://doi.org/10.15454/DKXVAC
*Rosaceae*	70–90	ARK (post-WGD)	9	8861	Strawberry, rose, peach, apricot, apple, pear	https://doi.org/10.15454/GUJBZB
*Cucurbitaceae*	25–50	ACuK (post-WGD)	22	17969	Melon, cucumber, gourd, watermelon, squash	https://doi.org/10.15454/A96TW6
Legumes	56–59	ALK (post-WGD)	16	13181	Peanut, lotus, chickpea, garden pea, barrel medic, pigeon pea, soybean, common bean, mung bean, adzuki bean, lupin	https://doi.org/10.15454/J9RN5S
*Solanaceae*	20–25	ASK (post-WGD 49 mya)	17	17879	Tomato, pepper, tobacco, sesame	https://doi.org/10.15454/TRBVMD

Summary of reconstructed ancestral angiosperm genomes listing the targeted botanical family, dating (in million years), the ancestral genome name (with WGD defining the delivered post-polyploidization ancestors in parentheses), number of protochromosomes, number of PGs, associated extant species involved and the link to the raw data information (README: description of the data provided in the table; ‘CONTACT’: person to contact for information on the data provided; ‘GENOME’: all versions of genomes used; ‘PHYLOGENY’: synteny information between chromosomes and derived ancestral chromosomes; ‘HOMOLOGY_GROUP’: number of conserved genes and corresponding conserved chromosomes)

### Data integration and query in SyntenyViewer

Previous synteny data are integrated into the SyntenyViewer tool relying on a DB with a Java web application for graphical dynamic web content processing, a web server (Apache HTTP and Apache Tomcat) and a PostgreSQL 9.6 instance to store the data (Figure 2). A spreadsheet-based data exchange format allows synteny data submission to SyntenyViewer, available at https://urgi.versailles.inrae.fr/Data/Synteny/Data-submission. It consists of a four-sheet file (in addition to a README that explains how to properly complete the whole file) regarding (i) the person to contact and authors of the data, (ii) genomic features (genes, position on chromosomes, annotation and genome versions) mainly from the Phytozome database (34), (iii) phylogenic relatedness between chromosomes of extant species and chromosomes of their inferred ancestors and (iv) homology groups that are used to store relationships between genes of several species, each gene being declared in the genome description sheet (see Point ii). Excel format is provided as an example for users to be completed, but text format is also possible for simplicity in the data submission process. An Extract-Transform-Load (ETL) toolbox, using open-source Talend Open Studio, is dedicated to validate the dataset consistency and completeness as well as its database insertion. This ETL is able to manage data updates on previously integrated datasets, by only inserting the changes, masking the previous versions and ultimately validating consistency and unicity of the data provided by users. Part of this validation step to avoid conflicts is handled into the ETL tool directly before integration into the database. The database itself relies heavily on unicity constraints over identifiers, some composite keys and some concatenations of entity (gene, dataset and chromosome) name and version. It contains several constraints used to ensure complete consistency over time between several integrations of updated versions. The version of the application sticks to the GnpIS information system (35). Dataset’s versions are displayed in the dataset form and detailed in the associated downloadable dataset from the RDG repository. Synteny data is then made publicly available when the format described on our website (https://urgi.versailles.inrae.fr/Data/Synteny/Data-submission), and the aims of SyntenyViewer are met. The usage of an all-in-one Excel file simplifies the data exchange between both parties. It also eases its manipulation by scientists for filling and submitting a unique file, which includes some static data extracted from the database (i.e. taxon scientific names), and hence guides the submitter with correct data at the beginning of the process and reduces the need for interactions between both parties. For data upload, the file can be provided through a ‘minimal web form’, allowing us to track the submission versions. Also, exchanges with the application maintainers (The Plant Bioinformatics Facility from URGI) are still possible by e-mail using the urgi-support@inrae.fr address.

### SyntenyViewer functionalities

There are two main entry points to visualize SyntenyViewer data (*cf* Supplementary Video). The first allows for selecting a dataset that provides gene conservation among a given botanic family or at a larger scale to whole monocot (grasses) or eudicot phylum, which then shows a query form described later. The other entry point allows for searching for a gene name (*via* its entire form or a prefix) across all datasets available. A search displays a popup with a short description of matching genes referenced in the database, and clicking on a selected gene loads the associated orthologous genes in the selected botanic family of interest, with an additional form for querying data in several manners as well as customizing display parameters. This query form offers users to enter the database through a gene ID, an extant or ancestral chromosome number and species of interest. The customized display parameters offer users to display windows with a specific number of (extant or ancestral) genes, to produce a compact view when having numerous genes visible, to swap gene order on chromosome or to hide chromosomes. Orthologous genes are given the same color code and are linked in a top-down manner between chromosomes, facilitating the identification of orthologous groups between genomes and species. Left clicking on a gene updates the synteny display centered on the selected gene. Right clicking a gene provides the associated gene information with its ID, its coordinates as well as links for redirecting toward numerous international databases (36, 37) following the Findable, Accessible, Interoperable, Reusable principles (38). Dedicated buttons make it possible to browse along the chromosomes. There is also a specific mode, which makes searches wider when no hit can be found for a specific gene on syntenic chromosomes: in such case, flanking genes are serially searched for orthologs on syntenic chromosomes until a first match is found, highlighting all relationships observed between all displayed genes of a given genomic region. At any stage, the SyntenyViewer’s Uniform Resource Locator is dynamically updated, and it can be bookmarked in the browser for sharing the visualization link, which makes users able to go back to previous work and pursue exploration. Finally, a download button, available after dataset selection, allows users to access its publicly available submission file along with relevant metadata (authors, description and DOI) through the RDG repository or through any link provided by the submitter. Data are downloadable by users in a tabular format from which additional visualizations can be performed such as dotplots using classical R packages available.

## Discussion

The delivered SyntenyViewer tool gives public access to validated and reviewed comparative genomics data either between angiosperm species or within major botanical families that can be used as a backbone to investigate evolutionary trends of genes, perform translational research of traits and conduct evolutionary developmental biology (for Evo-Devo) investigation of traits.

**Figure 3.**
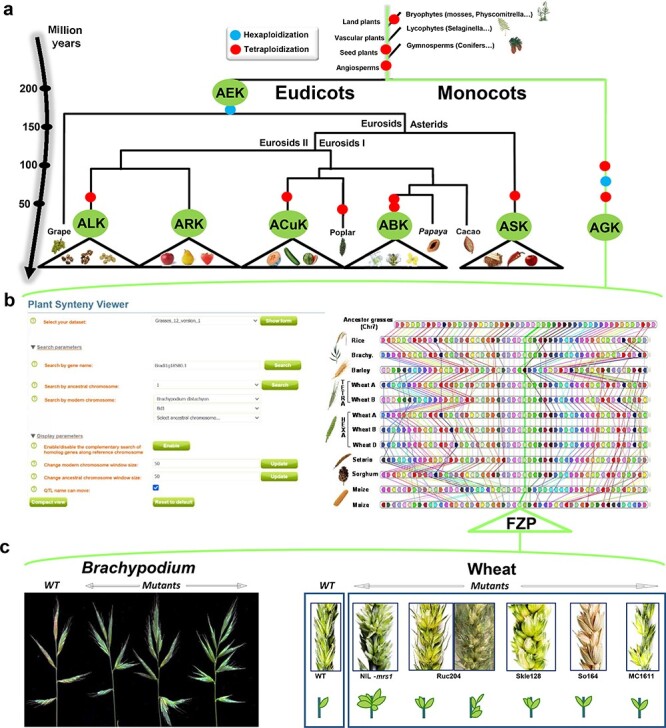
SyntenyViewer, a comparative genomics-driven translational research tool. (a) ‘Plant genome evolution from reconstructed ancestors’. The present-day angiosperm species (bottom) are represented along the evolutionary tree of the Angiosperms from founder ancestors (AGK, AEK, AcuK, ASK, ARK, ABK and ALK) of major botanical families with the time scale shown on the left (in million years). The polyploidization events that have shaped the structure of modern plant genomes during their evolution from inferred ancestors are indicated by red dots (duplication) and blue dots (triplication). (b) ‘SyntenyViewer screen capture’. SyntenyViewer tool with the setting parameters (search by gene name and ancestral or modern chromosomes) illustrated at the left and the derived comparative genomics data visualization, as detailed in the text, at the right (here for cereals). Genes are illustrated as colored boxes for each species (in lines), so that conserved genes are linked with colored lines between species. (c) ‘Synteny-based translational research of FZP gene in grasses’. FZP gene characterization in grasses with orthologs from SyntenyViewer (Panel b) and functional validation in wheat and Brachypodium (in mutants compared to wild type) in deriving similar SS phenotypes (adapted from (47)).

With the use of reconstructed ancestral genomes, structural (intron and exon structure) and functional Gene Ontology annotations of genes can be improved by comparing orthologous gene sets that may share similar (ancestral) genomic features. The reconstructed ancestral karyotypes can also be used to infer a parsimonious evolutionary model that assumes minimal numbers of genomic rearrangements (including duplications, inversions, deletions, fusions, fissions and translocations). SyntenyViewer allows deep investigation of evolutionary fates of ancestral genes/genomes, through precise identification of the changes involved (gains and losses of genes and associated gene ontologies) and their assignment to specific species or botanical families (Figure 3a). Among major evolutionary events, WGD can be investigated in detail as well as post-polyploidization partitioning between paralogous blocks forming ‘MF’ (also known as S for sensitive) and ‘LF’ (also known as D for dominant) chromosomal compartments (39, 40).

SyntenyViewer can also be used as a useful tool for translational research on genes driving key agronomical traits, particularly from model species (such as *Arabidopsis thaliana*) to crops (41). Such translational-based dissection of traits has been performed successfully in several botanical families, including legumes [for example, between *Medicato truncatula* and pea (42)] or grasses (Figure 3b) with *Brachypodium* used as a pivotal genome to dissect wheat traits (43) related to yield [i.e. NUE for nitrogen use efficiency (44)] or bread-making quality [GFC for grain fiber content (45), as well as carotenoid content (46)], among other cases. As a case example, Figure 3c illustrates the translational-based cloning of FRIZZY PANICLE (FZP) genes in bread wheat (47). Bread wheat inflorescences, or spikes, are characteristically unbranched and normally bear one spikelet per rachis node. From the gene conservation information delivered from SyntenyViewer, further validation needs to be conducted to establish the conservation of the phenotype or trait between the investigated species. In the case of FZP, based on wheat mutants with supernumerary spikelets (SS) and comparative genomics data between *Brachypodium* and wheat, it has been shown that the orthologous FZP gene, encoding a member of the APETALA2/ethylene response factor (AP2/ERF) transcription factor family, drives the SS trait in *Brachypodium*, bread wheat and rice (47, 48). Structural and functional characterization of the three wheat FZP homologous genes (WFZP-A-B-D of the allohexaploid bread wheat) revealed that coding mutations of WFZP-D cause the SS phenotype with the most severe effect when WFZP-D lesions are combined with a frameshift mutation in WFZP-A (47–49).

Beside translational research of genes, SyntenyViewer allows us to conduct ‘Evo-Devo dissection of traits’. SyntenyViewer allows us to compare a group of angiosperm species that acquired new phenotypes (or traits in the broad sense) in the course of evolution, compared to a group of species that did not acquire this trait. Following this strategy, comparing woody to herbaceous angiosperms allowed us to link the life history of trees to the amplification in tandem of genes involved in immunity, which has thus been proposed as a key process underpinning longevity of such a long lifespan species (50). This comparative Evo-Devo framework can be used to provide a better understanding of the molecular bases of major agronomical interest, such as seasonality (comparing annual *vs.* perennial species), photosynthesis (comparing C3 *vs.* C4 species) as well as grain and fruit developmental and quality traits in crops.

Overall, SyntenyViewer is a web-based tool delivering comparative genomics data either between angiosperm species or within major botanical families (including the *Rosaceae, Brassicaceae, Cucurbitaceae*, legume and *Solanaceae*) for evolutionary and translational research purposes.

## Supplementary Material

baad027_Supp

## Data Availability

All required links or identifiers are provided in the current article.
